# DNA damage regulation and its role in drug-related phenotypes in the malaria parasites

**DOI:** 10.1038/srep23603

**Published:** 2016-04-01

**Authors:** Devendra Kumar Gupta, Alok Tanala Patra, Lei Zhu, Archana Patkar Gupta, Zbynek Bozdech

**Affiliations:** 1 School of Biological Sciences, Nanyang Technological University, 639798, Singapore

## Abstract

DNA of malaria parasites, *Plasmodium falciparum*, is subjected to extraordinary high levels of genotoxic insults during its complex life cycle within both the mosquito and human host. Accordingly, most of the components of DNA repair machinery are conserved in the parasite genome. Here, we investigated the genome-wide responses of *P. falciparum* to DNA damaging agents and provided transcriptional evidence of the existence of the double strand break and excision repair system. We also showed that acetylation at H3K9, H4K8, and H3K56 play a role in the direct and indirect response to DNA damage induced by an alkylating agent, methyl methanesulphonate (MMS). Artemisinin, the first line antimalarial chemotherapeutics elicits a similar response compared to MMS which suggests its activity as a DNA damaging agent. Moreover, in contrast to the wild-type *P. falciparum*, two strains (Dd2 and W2) previously shown to exhibit a mutator phenotype, fail to induce their DNA repair upon MMS-induced DNA damage. Genome sequencing of the two mutator strains identified point mutations in 18 DNA repair genes which may contribute to this phenomenon.

Maintenance of genetic integrity is essential for proper functioning of a cell and is also vital for successful transmission of genetic information during the cell division. For this, eukaryotic cells developed an orchestrated DNA damage response encompassing distinct repair pathways for different types of DNA lesions^1^. These repair pathways are classified broadly into two categories: the excision repair pathways including nucleotide excision repair (NER), base excision repair (BER) and mismatch repair (MMR); and the double strand break repair pathways (DSBR) including homologous recombination repair (HR) and nonhomologous end joining (NHEJ)^2^. Given the complex life cycle of *P. falciparum,* the main causative agent of human malaria, which involves two hosts and numerous physiological/developmental stages, the parasite’s cellular DNA is under a constant exogenous and endogenous stress^3^. The major source of the genetic assaults in *Plasmodium* originates from replications errors, reactive oxygen species (ROS) generated during heme metabolism, and also free radicals generated by antimalarial drugs such as chloroquine and artemisinin^3^,^4^,^5^,^6^. Although, the majority of the genes of the DNA repair pathways are conserved in *Plasmodium*, until today, limited information exists about their regulation and specific function. Recently, it has been shown that *Plasmodium* parasites utilize predominantly the HR system to repair double strand breaks, which are the most deleterious forms of genetic aberrations and can lead to cell death^7^. On the other hand, the factors of canonical-NHEJ that are crucial for DSBR in most eukaryotes appear to be missing in the genome of the malaria parasites^8^. However, there is an experimental evidence for an alternative-NHEJ that has been suggested to repair double stranded breaks in the *Plasmodium* parasites^9^,^10^. This end joining process is believed to be guided by local microhomologies that are initially created by a non-processive DNA polymerase. This is followed by annealing, ligation and final repair in a process termed SD-MMEJ^11^. In *P. falciparum*, the DNA polymerase subunit β has been previously characterized^12^,^13^ and suggested to play the role in the SD-MMEJ presumably by creating the microhomologies^9^.

DNA damage recognition and repair occurs in the context of chromatin. In eukaryotes, chromatin plays a significant role particularly in regulating the DNA damage response^14^. During DNA damage, histone modifications and chromatin remodeling complexes alter the chromatin architecture to give access to the damaged site for the DNA repair enzymes^15^. In particular, H2A/H2A-X phosphorylation and H4 acetylations play significant roles in the recruitment of DNA repair factors such as ATM kinase, ATR kinase, Rad51, BRAC1, TP53, TIP60 HAT^16^,^17^. Most of the histone modifications such as acetylation, phoshorylation, methylation as well as several chromatin remodeling complexes, canonical in other eukaryotes, have been identified in *P. falciparum*^18^. However, the role of chromatin dynamics in the DNA damage response in *P. falciparum* remains unexplored.

Resistance of *P. falciparum* malaria to several classes of anti-malarial drugs such as chloroquine, pyrimethamine, sulfadoxine and more recently artemisinin had first emerged in Southeast Asia and South America and have (or it is expected to) subsequently spread around the world^19^,^20^,^21^,^22^,^23^. All drug resistance phenotypes have been associated with DNA polymorphisms; typically non-synonymous mutations in drug targets and/or drug transporters^24^,^25^,^26^. It has been suggested that a rapid emergence of the anti-malarial drug resistance phenotypes is linked with increased mutation capacity of some parasite strains (mutator phenotype), known as accelerated resistance to multiple drugs (ARMD)^27^. The ARMD parasites may harbor diverse genetic backgrounds which may provide additional advantage in developing the actual drug resistance mechanisms as well as alleviate a fitness cost^27^. Such mutator phenotypes were found in bacteria, yeast as well as mammalian cells^28^,^29^,^30^ and are typically associated with defects in the DNA replication machinery or DNA repair associated processes^31^. DNA mismatch repair deficient cells can be also unresponsive to several chemotherapeutic drugs such as etoposide, procarbazine, busulfan, cisplatin and doxorubicin^32^. Similarly, defective DNA repair machinery confers resistance to cisplatin and anti-parasitic drug monensin in other protozoa intracellular parasites *Trypanosoma cruzii* and *Toxoplasma gondii*.^33^,^34^. In malaria, it has been suggested that ARMD parasites have reduced DNA repair capacity as compared to the wild type (non-ARMD) parasites^35^. However, the underlying genetic and biochemical mechanisms responsible for the ARMD phonotype are still not understood.

Here, we characterized transcriptional responses of *P. falciparum* to DNA damaging agents in malaria parasite, to understand regulation of the parasite repair machinery. Using methyl methanesulphonate (MMS), we show that *P. falciparum* responds to DNA damage by upregulating specific genes of the DNA repair machinery and at the same time alters its chromatin structure. We also show that artemisinin, the primary anti-malarial drug, also induces DNA damage response (similar to MMS) involving both the transcriptional and epigenetic changes. Finally, *P. falciparum* ARMD strains seem to fail to induce these transcriptional and epigenetic responses to DNA damage which may underlay their increased mutational characteristics. Whole genome sequencing revealed several genetic polymorphisms in DNA repair genes that may contribute to the ARMD phenotype.

## Results

### Global transcriptional response to DNA damaging agents

**Figure 1:**
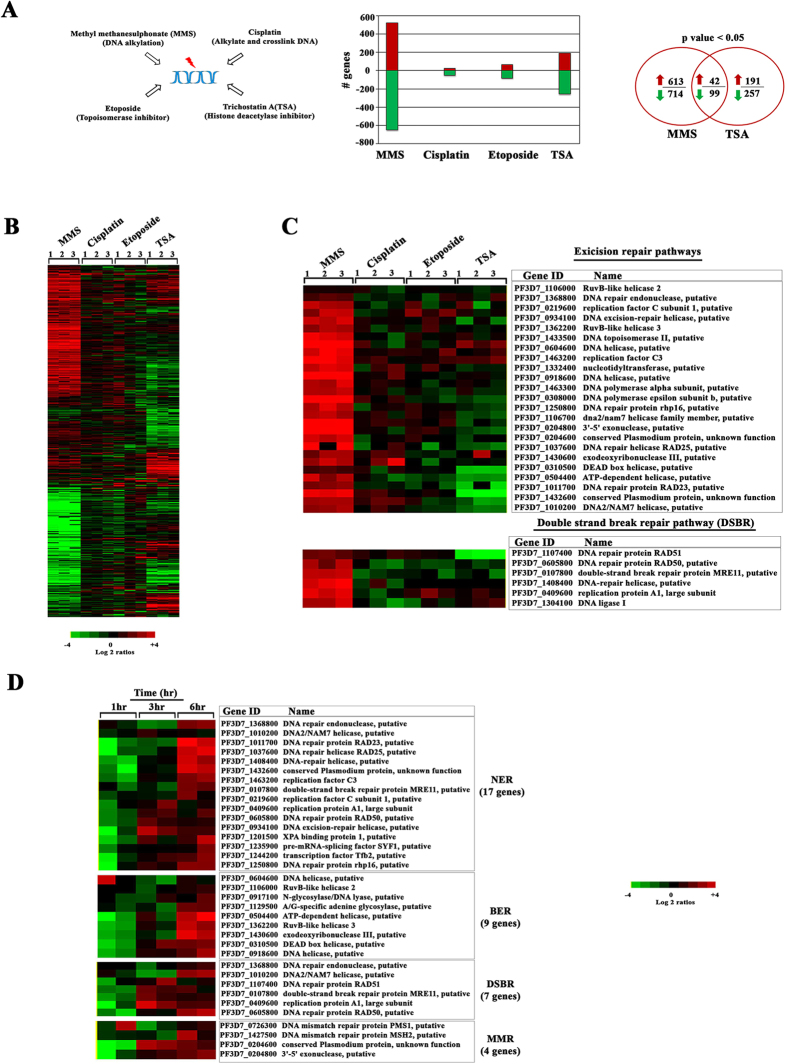
Global transcriptional response of *P. falciparum* to DNA damaging agents. (**A**) Four DNA and/or chromatin perturbation agents MMS (0.05%), Cisplatin (100 μM), Etoposide (100 μM), and TSA (50 μM) were chosen to study the DNA damage response of *P. falciparum* parasites. The bar graph represents the number of genes differentially induced by >2 fold after synchronized parasites were treated at the trophozoite stage for 6 hr. The Venn diagram represents differentially expressed genes between the MMS or TSA treatments. (**B**) Heat map represents up (red) and down (green) regulated genes induced by the drug treatments by >2-fold. The results are mean value of a relative mRNA abundance in three experimental replicates. (**C**) The expression pattern of 29 excision and 6 DSB repair genes which were up-regulated by MMS are shown along with the effect of cisplatin, etoposide and TSA among three independent replicates (p-value < 0.05). (**D**) The heat map represents differential expression of DNA repair genes in a time course experiment with samples collected at 1, 3 and 6 hr of the MMS (0.05%) treatment. Only with the 6 hr of exposure to MMS, DNA repair machinery was up-regulated with 9 BER, 17 NER, 4 MMR and 7 DBSR genes significantly up-regulated in the two independent replicates (p- value 0.0002–0.02).

The main goal of this study was to elucidate regulation of gene expression in response to DNA damage in the human malaria parasites *P. falciparum*. As the first step, we carried out genome wide gene expression profiling of *P. falciparum* exposed to several classes of DNA damaging chemical agents. Briefly, synchronized *P. falciparum* cultures were treated with MMS (0.05%), cisplatin (100 μM), etoposide (100 μM) and TSA (50 μM) for 6 hours (hr) at the trophozoite stage (28–32 hr post invasion, hpi). At these concentrations these agents are known to cause either DNA damage or otherwise interact with DNA in *P. falciparum*. MMS alkylates DNA bases and by that induces single and double strand breaks^36^,^37^. Treatment of *P. falciparum* with MMS (0.05%) is known to cause DNA damage (validated by the comet assay) and also to induce two DSBR genes, PfRAD51, PfRAD54^38^. Cisplatin is a DNA intercalating agent that inhibits DNA replication and transcription^39^ while etoposide inhibits topoisomerase II, which subsequently induces DNA damage^40^. In *P. falciparum,* 100 μM of cisplatin treatment for 6 hr caused sequence specific cisplatin-DNA adduct formation, which subsequently led to DNA damage-mediated parasite death^41^. 100 μM treatment of *P. falciparum* by etoposide was shown to induce chromosomal cleavage and altered the genetic integrity which was confirmed by pulse field gel electrophoresis^42^. For comparison, we also used a histone deacetylase HDAC inhibitor, trichostatin A (TSA) that was previously shown to have a profound effect on the chromatin architecture and gene expression in *P. falciparum*^43^,^44^. To measure global transcriptional responses we utilized the genome-wide *P. falciparum* DNA microarray as previously described^45^. The perturbation analyses for these drugs were done in triplicates and the transcription results were highly reproducible (Pearson correlation among biological replicates were, 0.6–0.8, p value < 0.05). Interestingly, MMS and TSA affected transcription of a considerable number of genes while cisplatin and etoposide had only moderate-to-low effect (Fig. 1a,b). In particular, expression of 1170 genes was altered by MMS by 2-fold from which 523 and 647 genes were up- and down-regulated, respectively. In contrast, cisplatin and etoposide altered expression of 79 and 146 genes by 2-fold, respectively. The effect of TSA on gene expression was similar to previously published results with differential expression of 448 genes^43^. There was a small but statistically significant overlap between genes induced by MMS and TSA with 42 and 99 being commonly up and down regulated, respectively (Fig. 1a right panel). This suggests that MMS and TSA effects on gene expression are unique to each drug.

Amongst the 141 commonly co-regulated genes by MMS and TSA, there is a significant enrichment of factors of host parasite interactions such as “merozoite invasion” and “PfEMP1 domain architecture” (data not shown). This is consistent with previous suggestions that such genes facilitate a general stress response that is not directly linked to the perturbation/agent mode of action^46^,^47^. MMS alone induced an additional set of genes that can be linked with indirect responses such as protein biosynthesis, palmitome proteins, ribosomal assembly genes, molecular chaperones, nuclear genes destined for mitochondria/apicoplast and glycolysis (Supplementary Fig. S1). Most of these functionalities could also represent indirect responses to the stress conditions. Nonetheless, one of the most predominant functional group induced by MMS was a set of genes associated with the DNA repair machinery that reflects directly its presumed mode of action. This supports previous studies showing that treatment of *P. falciparum* parasites with MMS (0.05%) for 6 hr results in DNA double strands breaks and up-regulation of two factors of the HR pathway PfRAD51, PfRAD54^38^. Here, we show that MMS induces a broad DNA damage response including most of the factors of both the excision repair machinery and DBSR (Fig. 1c). After the 6 hr exposure, there were 9 genes of BER, 17 genes of NER, 4 genes of MMR and 7 genes of DBSR significantly up-regulated in the two independent replicates (p value < 0.05) (Fig. 1d). This transcriptional induction requires at least 6 hr exposure of *P. falciparum* cells to MMS as shorter time exposures did not exhibit statistically significant enrichment of the DNA repair genes/pathways amongst the upregulated genes. However, for all four pathways there was at least one gene whose transcriptional induction occurred as early as 3 hr; including XPA binding protein 1 (PF3D7_1201500) of NER; putative DEAD Box helicase (PF3D7_0310500) of BER; replication protein A1, large subunit (PF3D7_0409600) of DSBR; and the conserved *Plasmodium* protein (PF3D7_0204600) of MMR. In future experiments it will be interesting to investigate if these genes might represent rate limiting steps in DNA repair induction in their respective pathways.

### DNA damage induces alterations in chromatin landscape

**Figure 2:**
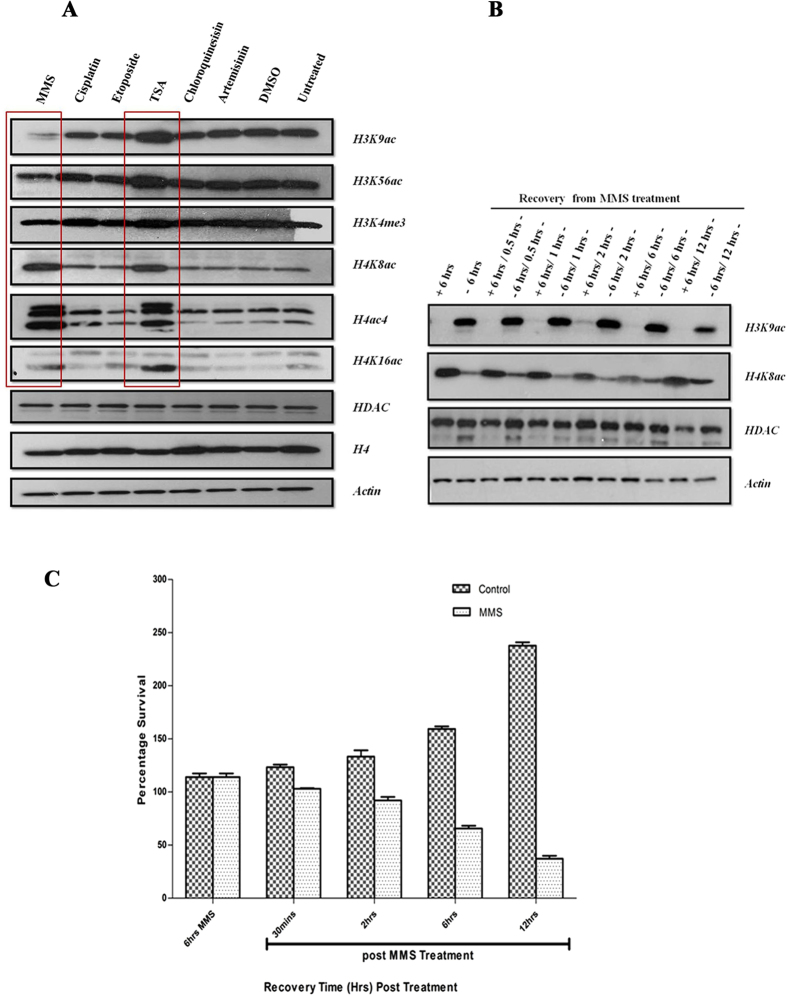
DNA damage affects the overall abundance of histone modifications during the *P. falciparum* IDC. (**A**) 3D7 parasites were treated with all drugs at the trophozoite stage for 6 hr and the overall abundance of individual histone modification was measured by western blotting. The MMS treatment resulted in reduction of H3K9ac and H3K56ac and increase of H4K8ac, H4K16ac and H4ac4. Conversely, TSA induced hyperacetylation of all studied H3 and H4 acetylations. Cisplatin and etoposide as well as artemisinin and chloroquine at IC-90 concentrations had no effect on the overall abundance on the histone modification compared to *P. falciparum* grown under normal condition or treated with the carrier solvent, DMSO. Specific antibody for HDAC enzyme, histone 4 and actin were used as loading controls. (**B**) Recovery dynamics of H3K9ac and H4K8ac were assessed by first treating with MMS for 6 hrs and subsequently drug was removed and samples were collected after 0.5 hrs, 1 hrs, 2 hrs, 6 hrs and 12 hrs. **(C**) Parasite survival after MMS (0.05%) treatment compared to untreated 3D7 (Control) parasites. Graphical representation shown is percentage survival in MMS treated and control parasites at 6hrs MMS and 30 mins, 2 hrs, 6 hrs and 12 hrs (post-MMS wash off treatment). The error bars represent SEM.

Chromatin alteration by histone modifications during DNA damage regulates the accessibility of DNA repair factors and other regulatory proteins^16^,^48^,^49^,^50^. The role of acetylation of histones 3 and 4 (H3 and H4) and phosphorylation of H2A/H2A-X in DNA damage response is well documented in yeast, mammals and more recently in *Toxoplasma gondii*^51^,^52^,^53^,^54^. Here, we wished to study the effect of DNA damage on histone modifications in *P. falciparum*. For that the trophozoite stage (28–32 hpi) parasites were treated with the same panel of drugs used for the transcriptional profiling with the same concentrations and duration of exposure (above). Simultaneously, trophozoite parasites were treated with inhibitory concentrations IC_90_ doses of artemisinin (50 nM) and chloroquine (70 nM). Subsequently, we utilized an immunoblotting analysis to study the abundance of several histone marks that were previously shown to play a role in the *P. falciparum* chromatin remodeling^55^. These included histone 3 Lysine 9 acetylation (H3K9ac), H3K56ac, H3K4me3 (lysine 4 trimethylation), H4K8ac, H4K16ac and H4Kac4 (K4, 8, 12, 16 acetylation) (Fig. 2a). Our results revealed that unlike cisplatin and etoposide, MMS, caused increases in acetylation at H4K8 and H4K16 and (likely) as a results of that at H4ac4. On the other hand, H3K9ac and H3K56ac were reduced after the 6hr MMS treatment. There were no detectable changes in the levels of the studied histone marks following treatment with IC_90_ doses of artemisinin and chloroquine. The increased levels of H4K8ac and the decreased levels of H3K9ac remained detectable even 12 hr after MMS was removed from the culture; past the 6 hr treatment (Fig. 2b). This may suggest that the repair in *P. falciparum* continues even after removal of MMS possibly in parasites in which the DNA damage was extensive that were beyond repair capacity. This is suggested by the fact that the majority of MMS treated parasite are dying gradually, diminishing the parasite population for as long as 12 hr post treatment (Fig. 2c). Interestingly, the studied histone modifications continue to function (possibly) as sensors/effectors of DNA repair, similar to other eukaryotes^16^. The MMS-induced effect on chromatin is distinct from TSA inhibition of HDAC that results in increases of H4K8ac, H4K16ac, H3K9ac and H3K56ac that was reproduced from our original study (Fig. 2a)^43^,^56^. The TSA-induced H4 and H3 hyperacetylations are reversible as early as 2 hr after drug removal^43^ which also contrasts the effect of MMS on chromatin remodeling.

### DNA damage induces genome wide spread of H4K8 acetylation whereas H3K9 acetylation preferentially marks the DNA repair genes

**Figure 3:**
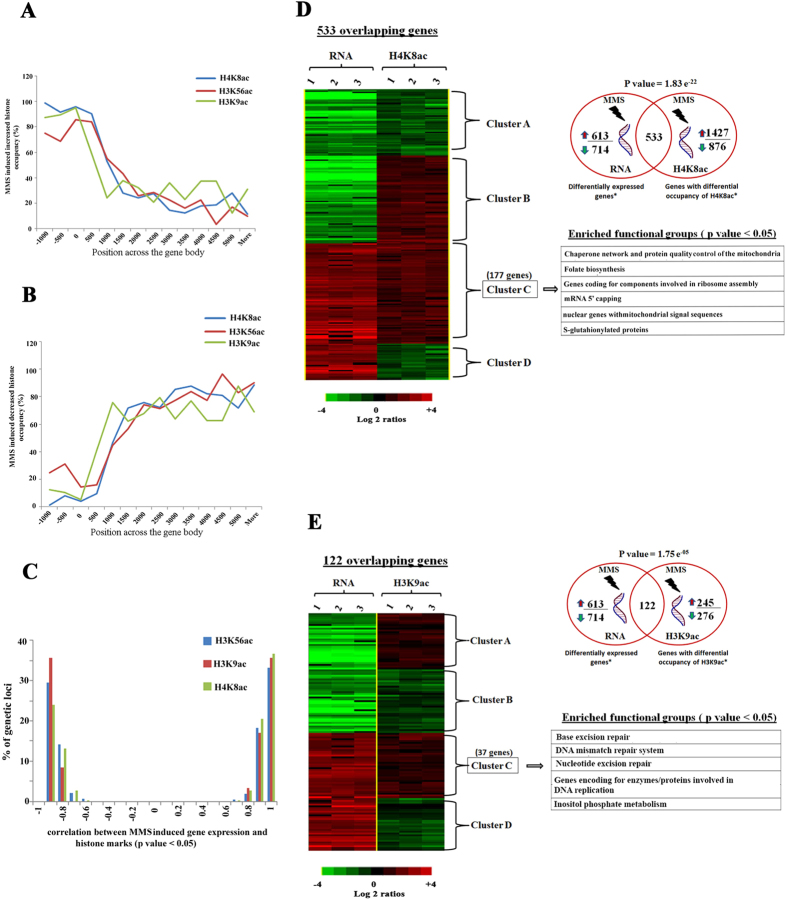
DNA damage induces genome wide spread of H4K8 acetylation whereas H3K9 acetylation preferentially marks the DNA repair genes. (**A**,**B**) The horizontal axis is the microarray oligonucleotide probes (MOE) position relative to the ATG start codon. The vertical axis is the number of histone marks associated genetic loci normalized to the number of probes which represent that particular region on the genome and these genetic loci are those which have increased (**A**) or decreased (**B**) ChIP signal in presence of MMS (p value < 0.05). (**C**) The correlation between MMS induced gene expression and histone marks is shown. (**D**,**E**) Association between differential expression and altered histone marks occurrence during MMS treatment is shown. To study such association, we correlated ChIP-on-chip dataset and RNA expression dataset obtained from MMS treated trophozoite parasites and it results in significant overlap between up-regulated/down-regulated genes (>2 fold, p value < 0.05) due to MMS treatment and with those that had altered histone marks (p value < 0.05). For H4K8ac (**D**) and H3K9ac (**E**), we identified 533 and 122 overlapping genes. Hierarchical clustering and enriched functional groups of these 533 and 122 genes were also shown, which are divided into 4 clusters depending upon on correlation between ChIP-on-chip and transcriptome dataset.

In the next step, we focused on the two predominant histone marks H3K9ac and H4K8ac, and carried out chromatin immunoprecipitations coupled with DNA microarray (ChIP-on-chip). The main objective was to identify chromosomal regions associated with H3K9ac, H4K8ac and H3K56ac that are responsive to DNA damage. Hence, trophozoite stage parasites (28–32 hpi) were treated with 0.05% MMS for 6 hr and ChIP-on-chip was performed as described^55^ (also see Materials and methods). The total number of genetic loci (defined by the microarray oligonucleotide probes) which were differentially acetylated as a result of MMS (induced/reduced) for H4K8ac, H3K9ac and H3K56ac were 2689, 528 and 2527, respectively (p value < 0.05). From these, 60% (1,592), 46% (243) and 52% (1,318) of genetic loci displayed a significant increase in H4K8ac, H3K9ac and H3K56ac occupancy in the presence of MMS. Crucially, MMS-induced H4K8ac, H3K9ac, H3K56ac appears to be enriched within intergenic regions; upstream of protein coding regions (Fig. 3a). In contrast, loss of acetylation was observed within the coding regions with progressively decreasing occupancy towards the 3′ termini of the genes (Fig. 3b). It has been previously shown that endogenous H4K8ac is predominantly enriched at the intergenic regions, whereas H3K9ac and H3K56ac are localized mainly within the 5′ regions of the coding regions^55^. Our result suggests that the MMS mediated DNA damage induces acetylations at regions that overlap with genetic loci that are already associated with these histone marks at putative promoter regions during the *P. falciparum* life cycle under normal growth conditions.

To study the effect of the MMS induced histone modifications on transcription, we correlated the ChIP-on- chip and RNA expression results. Interestingly, there was a sharp bimodal distribution with positive and negative correlations between MMS induced gene expression and histone acetylations (p value < 0.05) (Fig. 3c). In particular, there were 533 genes whose altered transcription (>2 fold, p value < 0.05) correlated significantly with alterations in occupancy of H4K8ac (p value 1.83E-22, hypergeometric test). From these there were 177 genes in which the increase in H4K8ac coincided with transcriptional up-regulation. Pathway enrichment analyses identified that these genes belong to several cellular processes of protein metabolisms including ribosome assembly, chaperones/protein folding, as well as other biosynthetic pathways including mitochondria (“*metabolic nuclear genes destined to mitochondria*”) and folate biosynthesis (Fig. 3d). In yeast, MMS induces up-regulation of chaperones and also facilitates trafficking of proteins into mitochondria, which constitutes the indirect responses to DNA damage^57^,^58^. Our results suggest that this process is evolutionarily conserved and that some of the indirect transcriptional responses induced by MMS are linked with H4K8ac. For H3K9ac, we identified 122 genes (132 genetic loci, p value 1.75E-05, hypergeometric test) whose abundance correlated with expression. Out of the 122 overlapping genes, 37 show positive correlation (p value < 0.05). Strikingly, this gene set is enriched for factors of the DNA repair and DNA replication associated pathways (Fig. 3e). In mammals, it is the H3K56ac which typically regulates the expression of the DNA damage repair genes under stress conditions^59^. However, our results show that in *P. falciparum*, the occupancy of H3K56ac is not linked with DNA repair machinery but instead with genes encoding S-Glutathionylated and mitochondrial proteins (Supplementary Fig. S2). Hence, our results suggest that *Plasmodium* may have undergone an evolutionary diversion, where regulation of DNA repair was “taken over” by H3K9ac. Intriguingly, this occurs despite the fact that MMS causes an overall reduction of H3K9ac levels in *P. falciparum*. In future studies, it will be interesting to explore the molecular mechanisms that facilitate this chromatin-linked induction of the DNA damage repair genes.

### Artemisinin induces DNA damage response in *P. falciparum*

**Figure 4:**
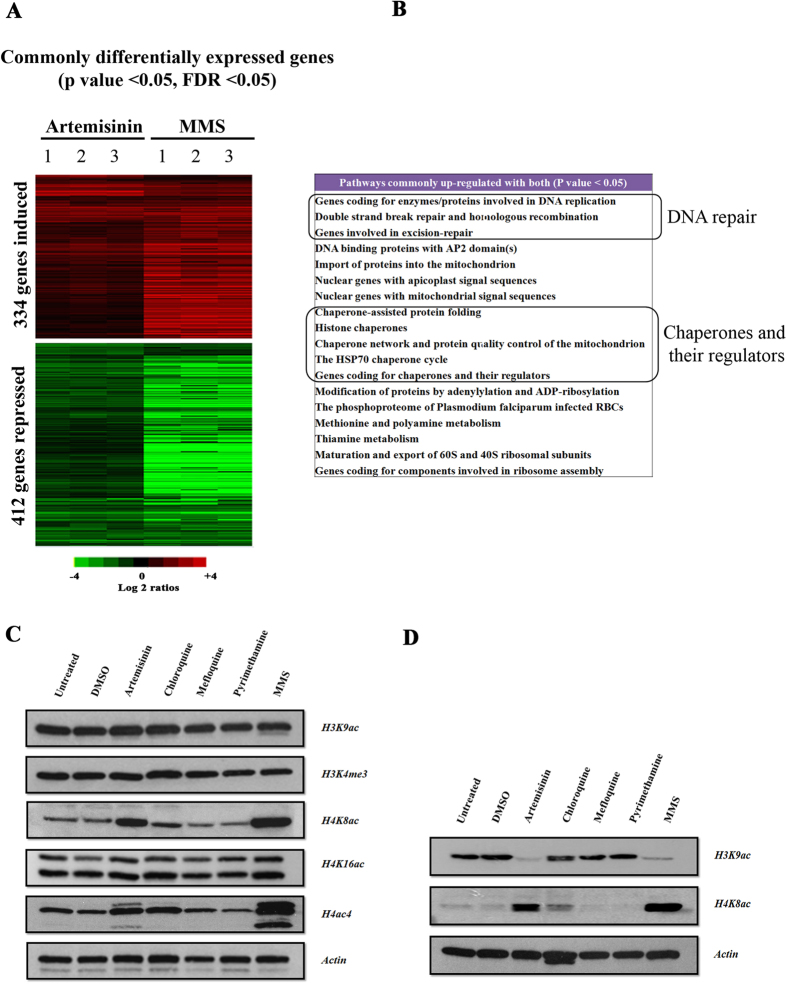
Artemisinin induces DNA damage response in P. falciparum. (**A**) The synchronized *P. falciparum* cells were treated with clinical doses of artemisinin (1 μM) and MMS (0.05%) for 6 hr at the trophozoite stage. Genes with similar differential transcription for both MMS and artemisinin are shown in the heat map. The experiments were done in triplicates. (p value < 0.05, FDR < 0.05) (**B**) Pathways, which are commonly up-regulated by both artemisinin and MMS. Schizont (**C**) and Trophozoite stages (**D**) of parasites were treated with clinical doses of different anti-malarial drug for 6 hr: artemisinin (1 μM), chloroquine (30 μM), mefloquine (5 μM) and pyrimethamine (5 μM) and with MMS (0.05%). Immunoblot analyses were carried out using primary antibodies probed against the core histone modifications.

Artemisinin is the most potent antimalarial drug which has been established as the first line treatment of multiple drug resistant strains of *P. falciparum*^60^. Although, artemisinin have been shown to induce a DNA damage response in cancer cells, its effect on the *Plasmodium* genomic DNA remains debatable^6^. Artemisinin may induce DNA damage in malaria parasite giving positive results of an *in vitro* comet assay^61^. In order to provide evidence for an artemisinin role in DNA damage, we wished to investigate similarities between artemisinin and MMS in terms of their effect on the DNA repair machinery in *P. falciparum*. For this the 3D7 strain of *P. falciparum* was treated at the trophozoite stage (28–32 hpi) with 1 μM of artemisinin for 6 hr. We chose the physiologically relevant dose of 1 μM which corresponds to plasma concentrations of the drug when administered to patients^62^. Hierarchical clustering of RNA expression data identified 334 and 412 genes that were commonly up- and down-regulated, respectively, by both MMS and artemisinin (p value < 0.05, FDR < 0.05) (Fig. 4a). Functional enrichment analysis revealed that in addition to several biological processes associated with protein metabolism and DNA replication, at the given treatment conditions, artemisinin also induces genes of the DNA repair pathways (Fig. 4b). These include 8 genes of excision repair and 4 genes of DSBR machinery. Interestingly, Rad50 and MRE11 which constitute the MRN exonuclease complex which are regarded as DNA damage sensors of DSBR were also up-regulated. Moreover, Rad51, which is another important repair protein of DSBR pathway, was also up-regulated by artemisinin. Rad51 is a key nucleoprotein, which is required for strand invasion and strand annealing steps of DSBR^38^. It has been also reported that artemisinin inhibits topoisomerase enzymes in cancer cells, which leads to generation of single and double strand breaks and subsequently to cell death^6^. Interestingly, artemisinin treatment in the parasite also leads to under-expression of two DNA topoisomerases: PF3D7_1433500 and PF3D7_1365600. Moreover, artemisinin has an effect on the overall abundance of H3K9ac, H4K8ac and H4ac4; similar to MMS (Fig. 4c,d). This contrasts the lower dose of artemisinin IC90, which does not affect these histone modifications (see Fig. 2). Specifically, at 1 μM, artemisinin induced H4K8ac in the trophozoite and schizont stages and suppressed H3K9ac in the trophozoite stage but not at the schizont stage. In addition to artemisinin, we tested physiologically relevant concentrations of three antimalarial drugs including chloroquine, mefloquine and pyrimethamine and showed no effect on histone acetylation. The only exception is chloroquine that caused a moderate increase of H4K8ac in schizonts. Taken together, DNA damage is likely a part of artemisinin antimalarial mode of action given the fact that its effect on *P. falciparum* includes inductions of the DNA damage repair mechanisms and histone acetylations in the similar fashion to MMS; presumably as a result of DNA damage.

### ARMD parasites displaying mutator phenotype have defective DNA repair

**Figure 5:**
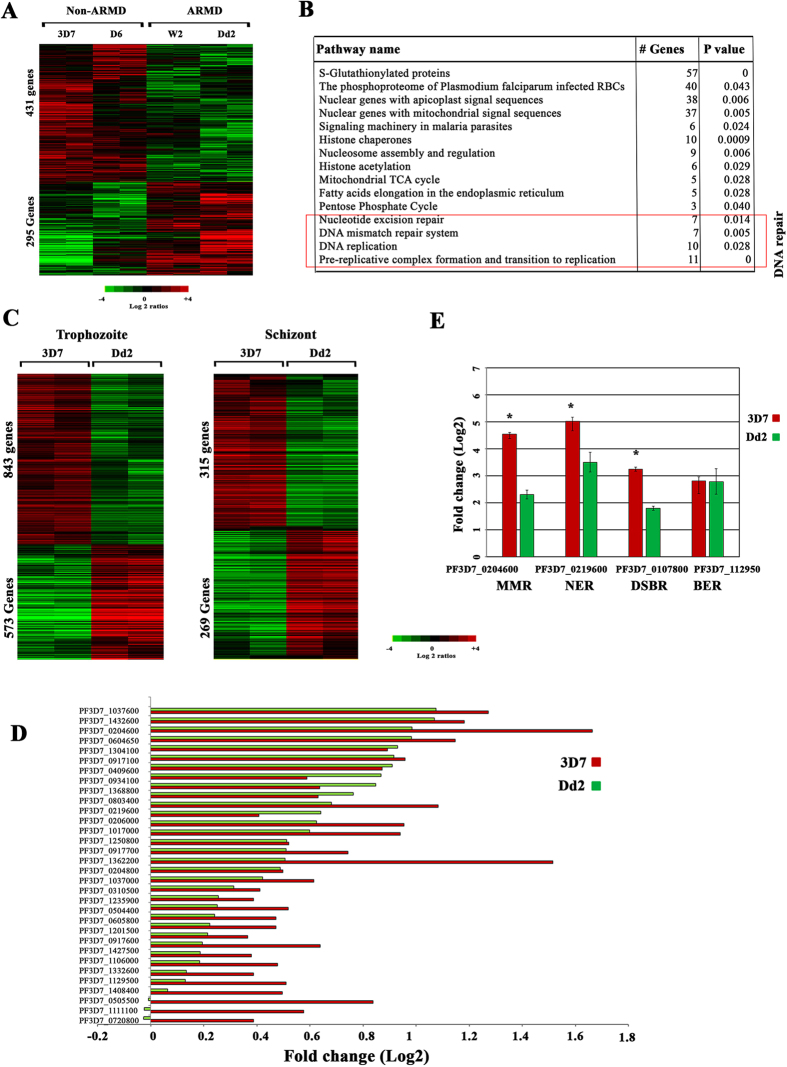
ARMD parasites displaying mutator phenotype have defective DNA repair. (**A**) *P. falciparum* ARMD and non-ARMD parasites were treated with MMS (0.05%) at trophozoite stage for 6 hr. Heat map showing the MMS induced differentially expressed genes between the ARMD and non-ARMD parasites (p value < 0.05) at trophozoite stage. Shown are the mean-centered expression log2 ratios for these genes. (**B**) Functional enrichment analysis on the 431 up-regulated genes is shown. (**C**) The gene expression patterns of differentially expressed genes at trophozoite and schizont stage are shown between 3D7 and Dd2. (**D**) 34 repair genes, which were differentially expressed, were plotted for ARMD (Dd2) and non-ARMD (3D7). (**E**) Validation of differential expression of genes between 3D7 and Dd2 was done by Quantitative RT-PCR. Quantitative RT-PCR was done on four genes using RNA derived from samples treated with MMS for 6 hr. PF3D7_0204600 belongs to mismatch repair (MMR), PF3D7_0219600 belong to nucleotide excision repair (NER), PF3D7_0107800 belong to double strand break repair (DSBR) and PF3D7_112950 belongs to base excision repair (BER). Relative fold change in RNA expression was computed by 2^−∆∆Ct^ method. The reference control gene used was PF3D7_1218600 (arginyl-tRNA synthetase). Error bars shows the standard deviation of the 2^−∆∆Ct^ value over triplicates. (*represents genes which were differentially expressed between 3D7 and Dd2).

It had been previously suggested that defective DNA repair may contribute to the *P. falciparum* “mutator” phenotype termed ARMD (accelerated resistance to multiple drugs)^35^,^63^. To elucidate the underlying mechanism of the ARMD phenotype, we studied differences in transcription and chromatin structure between ARMD and non-ARMD parasites under DNA damaging conditions. For this, we treated two ARMD (Dd2 and W2) and two non-ARMD parasites strains (3D7 and D6) with MMS (0.05%) for 6 hr at the trophozoite stage (28–32 hpi). The two ARMD *P. falciparum* strains, Dd2 and W2, originate from Southeast-Asia (Indo-China) and display higher frequencies in acquiring drug resistance *de novo* under relatively short drug selection intervals^27^. In contrast, the non-ARMD strain 3D7 and D6 originate from “Netherland” and “Sierra Leone” and can remain drug susceptible even after prolonged pressure with anti-malarial drugs^27^. Overall, MMS was able to modulate expression of 1,065 1,018 1,093 and 1,014 genes in W2, Dd2, 3D7 and D6, respectively (>2 fold, p value < 0.05). From these, we identified 431 genes that were overexpressed in both non-ARMD and 295 genes overexpressed in both ARMD parasites simultaneously (>1.5 fold, p value < 0.05) (Fig. 5a). Results of a functional enrichment analysis of the 431 genes show a similar representation of physiological processes detected by our initial transcriptional perturbation study of the 3D7 strain (Fig. 5b). This suggests that the MMS effect on transcription is robust amongst nonisogenic strains of *P. falciparum*. As expected, genes of both MMR and NER repair machinery were found to be significantly up-regulated in these non-ARMD parasites. However, these genes were not induced by MMS in the ARMD parasite strains in both the trophozoite (24–28 hpi) and schizont (34–38 hpi) stages (Fig. 5c). Fig. 5d represents the microarray based signal for 34 DNA repair genes that were differentially induced by MMS in 3D7 but show no (statistically insignificant) changes in their expression in the Dd2, ARMD strain. Subsequently, differential expressions of 4 genes were validated by quantitative RT-PCR. These were: PF3D7_0204600 of MMR, PF3D7_0219600 of NER, PF3D7_0107800 of DSBR and PF3D7_112950 belongs to BER (Fig. 5e). These results suggest a failure to response to DNA damage by sufficient upregulation of DNA repair mechanisms may be one of the underlying mechanisms of the ARMD phenotype. This is however not linked with the changes in the histone modification that are responsive to MMS in all tested *P. falciparum* strains (Supplementary Fig. S3).

In yeast, bacteria as well as mammals, mutations in DNA repair genes underlay mutator phenotypes, which can subsequently result in rapid emergence of drug resistance^28^,^29^,^30^. Here, we carried out whole-genome sequencing using Illumina MiSeq platform of the four strains used in these experiments: ARMD (Dd2, W2) and non-ARMD (3D7, D6). For this, genomic DNA was harvested immediately after the transcriptomics study (less than 10 generation) in order to avoid accumulation of neutral mutations during normal grow in an *in vitro* culture as described^64^. Compared to the 3D7 reference strain, we identified 6,550, 7,231 and 7,253 non-synonymous SNPs within coding regions of 2099, 2,089 and 2,046 genes in the Dd2, W2 and D6 strains, respectively (Supplementary Table S1). Focusing on exonic non-synonymous SNPs in DNA repair genes, total 57 of these were polymorphic in at least one strain (Supplementary Table S2). From these, there are 18 DNA repair genes with SNPs found exclusively in the ARMD parasites (Table 1).

**Figure 6:**
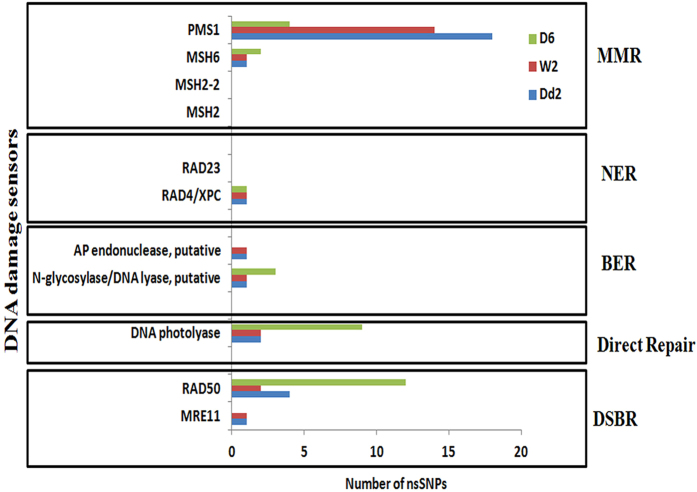
DNA polymorphism in DNA damage sensors between ARMD and non-ARMD parasites. There are three different repair mechanisms: DNA excision repair (BER, MMR, NER), double strand break repair (DSBR) and direct repair (DNA photolyase). Each repair mechanism has different set of sensory proteins which detect DNA damage, for example RAD23 and XPC are the DNA damage recognition proteins for NER repair mechanism. Each box have the distribution of SNP in DNA damage sensory proteins for a specific repair pathway among D6, 3D7 (non-ARMD) and Dd2, W2 (ARMD). Also, included amino acid mutation which changed form reference.

Four of the DNA repair genes have been previously reported to carry same mutations and were found to be associated with artemisinin resistance in Southeast Asia^65^. These are PF3D7_0509500 (conserved *Plasmodium* protein, unknown function), PF3D7_0710100 (conserved *Plasmodium* protein, unknown function), PF3D7_1106000 (RuvB-like helicase 2) and PF3D7_1343400 (DNA repair protein RAD5, putative)^65^,^66^. In addition, there were also ARMD-specific polymorphisms in genes encoding “sensors” of DNA damage (Fig. 6). This includes homologues of MRE11 (PF3D7_0107800) and AP lyase (PF3D7_0305600) belonging to DSBR and BER pathway, which showed polymorphisms in the ARMD strains compared to non-ARMD. Also, we identified extensive polymorphism in *P. falciparum* homologue of PMS1 protein (PF3D7_0726300) which potentially involved in MMR repair. Interestingly, artemisinin resistant parasites from Southeast Asia were found to carry nonsynonymous SNPs in the PMS1 gene^65^. Although, further experimental studies are needed to investigate the role of these mutations in the ARMD phenotype of *P. falciparum*, here we showed their occurrence in the parasites cells that failed to respond to DNA damage induced by MMS.

## Discussion

**Figure 7:**
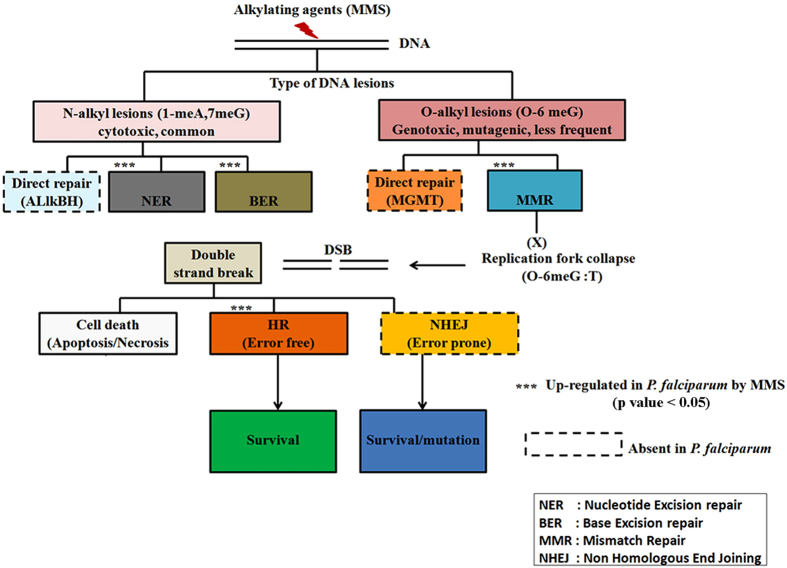
Overview of the *P. falciparum* DNA damage pathways and their transcriptional properties under MMS-induced stress. The initial N- and O-alkyl lesions of *P. falciparum* DNA caused by MMS leads initially to transcriptional induction of nucleotide excision repair (NER), base excision repair (BER) and mismatch repair (MMR). The subsequent induction of the double strand break repair (DSBR), namely the homologous recombination repair (HR), suggests that the MMS induced O-alkyl lesions was not be fully repaired by MMR. The transcriptional induction encompasses the majority (if not all) components of the *P. falciparum* DNA damage repair. Other components of eukaryotic DNA repair pathways such as nonhomologous end joining (NHEJ), or enzymes of direct repair (ALlkBH and MGMT) are not present in the *P. falciparum* genome (dashed line outline). Although extensive DSB could lead to cell death, 6 hr treatment with 0.05% MMS was likely counteracted by the DNA repair factors and did not lead to upregulation of putative factors of *P. falciparum* cell death. ***indicates statistically significant functional enrichment of genes of particular pathway amongst genes upregulated as a result of MMS-induced DNA damage. This outline is based on bioinformatics sequence analysis and the transcriptional results presented in this study.

The DNA repair machinery as well as the overall DNA damage response in *P. falciparum* are believed to contribute to many crucial biological functions of malaria parasites including antigenic variation and copy number variation^3^,^67^. Hence, full characterizations of these processes will have a great value for understanding of general biology of the *Plasmodium* parasites. Here, we demonstrated that when challenged with a DNA damaging agent, *P. falciparum* cells respond by broad transcriptional changes (21% of the genome) which include up-regulation of both DSBR and excision repair pathways. Similar type of responses were reported in yeast, in which alkylating agents induce differential expression in 1/3 of the yeast genome^68^. Like in yeast, in *P. falciparum*, DNA damage leads to upregulation of genes involved in generic (indirect) stress responses such as protein synthesis and turnover as well as mitochondrial functions but also direct responses, involving genes of the DNA repair machinery. Many of the canonical DNA repair genes are conserved in the *Plasmodium* genome and in this study we are providing transcriptional evidence of their functional involvement. This include upregulation of enzymes that are believed to sense a DNA lesion and initiate specific repair pathways as a result^69^. In *P. falciparum,* these include the homologues of A/G specific adenine glycosylases, XPC-h23B, mutS heterodimer (MSH2-MSH6) and MRN exonuclease complex (MRE11-Rad50) which corresponds to DNA damage sensors for BER, NER, MMR and DSBR, respectively (Supplementary Table S3). Besides the sensors, MMS was previously shown to induce overexpression of three *P. falciparum* DSBR genes, PfRAD51, PfRAD54 and PfRPA1^38^. Here, we show that the MMS-mediated DNA damage results in induction of the entire DNA repair machinery present in the *Plasmodium* genome (Fig. 7). Although, a single alkylating agent can generate different types of DNA lesions; each DNA lesion is recognized and repaired by a distinct pathway^70^. The two most common DNA adducts generated by MMS are: N7-methyl guanine (7 me G) and O6-methyl guanine (O6 me G)^37^. N-alkyl lesions are mostly repaired by BER, NER and direct repair enzyme AlkB homologue, whereas O-alkyl lesions are commonly repaired by MMR and a distinct direct repair enzyme, known as MGMT^70^,^71^. Particularly, O-6meG has higher tendency to form mispairs with thymine following replication^71^. The O-6 meG:T mispairs are recognized by mutS (MSH2-MSH6) which leads to activation of the MMR pathway^72^. If O6-meG:T mispairs, remains unrepaired, it leads to a futile cycle which results in the collapse of the replication fork and subsequently induction of double strand breaks^37^. This leads either to apoptosis (if the damage is too extensive) or the double strand breaks are repaired by either HR/NHEJ pathway^37^,^70^,^71^. Our results demonstrate that in *Plasmodium*, MMS treatment induces excision repair (NER, BER, and MMR) and DSBR (HR). As expected NHEJ and/or several unique direct repair enzymes (MGMT, AlkBH) do not play a role as most of their components are missing in the *Plasmodium* genome^8^,^73^. The DNA polymerase subunit β (PF3D7_0625300) the putative factor of SD-MMEJ, was not up-regulated by MMS which does not exclude this gene from DNA repair but indicate its distinct responsive properties to DNA damage.

In other eukaryotic cells, DNA damage can cause an arrest of the cell cycle and subsequently programmed cell death (reviewed in^74^,^75^,^76^). Analogously, exposure of *P. falciparum* to MMS leads to deceleration of the IDC (see below) and subsequent death even after MMS removal (Fig. 2c). In particular, after 6 hr exposure of *P. falciparum* schizonts to MMS, the overall transcriptome correlated with the 30 hr post invasion (hpi) rather than 38 hpi that was exhibited by the untreated cells (Supplementary Table S4) (for details about the correlation-based hpi estimation see^77^). This indicates that the IDC progression was arrested at the initial stage of the treatment and that thus the MMS induced transcriptional changes are related to DNA damage either as a direct response or as a consequence of this IDC slowdown. This is similar to transcriptional responses to other small molecular agents^47^. Nonetheless, there are two lines of evidence that the upregulation of DNA repair genes represents a direct response to the DNA damage; (i) the upregulation occurs at multiple IDC stages (trophozoites and schizonts) in which MMS otherwise deregulates different sets of genes; (ii) the DNA repair genes are not upregulated in the ARMD parasites that exhibit an IDC arrest identical to the Non-ARMD strains otherwise (for both see Fig. 5).

Our results also illustrate that MMS mediated DNA damage leads to considerable changes of histone modification, specifically H4K8ac, H3K9ac and H3K56ac. In yeast and mammals, DNA damage response is initiated by hyperacetylation of H4 by MYST histone acetyltransferase (MYST-HAT)^49^. It was also shown that mutations in any of the four H4 lysine residues impedes DNA repair in yeast^78^. In agreement with this, the *Plasmodium*, PfMYST-HAT acetylates H4K5, 8, 12 and 16 and its overexpression enhances protection from DNA damaging agents MMS and campthothecin^79^. This suggests that the histone 4 role in DNA damage is evolutionary conserved in the malaria parasites. The ChIP-on-chip results revealed that MMS induced H3K9ac and H4K8ac are predominantly enriched in the intergenic regions and thus are possibly associated with transcription (Fig. 3c). This is in contrast with histone deacetylase inhibitors such as apicidin and TSA that show very limited positive correlation between gene expression and altered acetylation induced by their effect in *P. falciparum* as well as mammals^56^,^80^. In particular, MMS-induced H4K8ac occurs at the transcriptionally up-regulated genes that constitute a putative generic response to DNA damage (protein turnover, mitochondria). Conversely, the increase of H3K9ac occurs preferentially at DNA repair genes that are also induced as a result of MMS-mediated DNA damage. Under normal growth condition, H3K9ac and H4K8ac are selectively enriched on actively transcribed genes belonging to metabolism and ribose assembly related functions in *P. falciparum*^55^. In the future, it will be important to investigate the molecular mechanism of H4K8ac and H3K9ac dynamics in transcriptional up-regulation during DNA repair.

Artemisinin appears to induce DNA damage in *P. falciparum* parasites presumably as a result of oxidative stress generated by this drug^81^. This is analogous to cancer cells where artemisinin engages DSBR^82^,^83^. Here, we observed that at a physiologically relevant dose (1 μM), artemisinin elicits similar transcriptional and epigenetic response that is induced by MMS. Importantly, both agents induced transcriptional up-regulation of parasite excision repair and DSBR. Additionally, we observed that both agents can induce expression of genes imported to the mitochondria/apicoplast. This is consistent with the results in yeast where DNA damage results in import of many proteins into mitochondria from nucleus, which includes pro-apoptotic proteins such as p53, as well as several DNA repair and cell cycle regulatory proteins^84^,^85^. Previously, Natalang *et al*. has shown that 3 hr exposure to 780 nM of artemisinin treatment at trophozoite stage altered transcript abundance of 398 genes none of which is involved in DNA repair^46^. Here, we showed that 6 hr treatment of *P. falciparum* cells by both MMS and artemisinin (1 μM) is needed before the gene expression and histone modification characteristics of DNA repair is detectable. Only then the DNA repair machinery is upregulated together with other ~1,500 genes (p value < 0.05). In natural infections, artemisinin reaches up to 1 uM concentrations in ~40 min and then it is rapidly eliminated with a half-life of ~20 min^86^. This pharmacokinetic profile may or may not provide sufficient time for artemisinin to cause DNA damage. Intriguingly, the kelch-13 protein (PF3D7_1343700) that has been associated with artemisinin resistance^87^ was upregulated in our artemisinin perturbation experiments (p value = 0.0004). Also, several factors of the DNA repair machinery have been linked with artemisinin resistance *in vivo* and *in vitro*^65^,^66^. The results here open a plausible possibility that DNA damage contributes to the “real-life” artemisinin mode of action and with that the artemisinin resistance phenotype that is presently spreading through Southeast Asia^23^,^87^,^88^.

Defects in DNA repair machinery were demonstrated to play significant roles in mutator phenotypes in various organisms such as yeast, mammals, bacteria, *T. brucei* and *T. gondii* 29,30,33,34,89. Here, we showed that the ARMD (mutator) phenotype of *P. falciparum* may be underlined by differential transcriptional regulation of genes involved in the DNA repair machinery when the parasites are under a DNA damaging stress. In particular, MMS was able to induce both MMR and NER repair machinery in the non-ARMD parasites but failed to up-regulate these genes in the ARMD parasites. This is consistent with the previous results, which suggested that defective DNA repair may contribute to the emergence of the ARMD parasites in Southeast Asia^35^,^63^. Although in our experiments, we did not link this phenomenon with (any) changes in histone modifications, further careful investigations are needed to see if the ARMD phenotype also involves epigenetic factors. Lastly, polymorphism in 18 DNA repair genes may lead to this impaired DNA repair functions. This suggestion is based on the fact that point mutations in mismatch repair genes leads to defective DNA repair which confers mutator phenotype in yeast and bacteria^29^,^89^. Further studies need to be done in order to understand the mechanism of how does mutation in DNA repair genes regulates the development of mutator phenotype in malaria parasites.

## Material and Methods

### Parasite culture and drug treatments

*P. falciparum* strains (3D7, Dd2, W2, and D6) were cultured using standard protocol for continuous culturing^90^. Drug treatments were carried out in sorbitol-synchronized trophozoite and schizont stage. For all drug treatment experiments, parasite culture was kept at 5% parasetemia and 2% hematocrit. Transcriptional profiling and immunodetection analysis were carried out at trophozoite stage in 3D7 under identical conditions with following drugs: MMS (0.05%), cisplatin (100 μM), etoposide (100 μM) and TSA (IC90/50nM). The four antimalarial drugs studied were artemisinin (1 μM), chloroquine (30 μM), mefloquine (5 μM) and pyrimethamine (5 μM).

### RNA isolation and cDNA synthesis

For MMS (0.05%) induced transcriptional analysis, synchronized parasites were harvested after 1, 3 and 6 hr of treatment, whereas it was only one time point of 6 hr of treatment for cisplatin (100 μM), etoposide (100 μM) TSA (IC90/50 μM) and artemisinin (1 μM). Before RNA extraction, drug treated parasites were washed with PBS twice to obtain infected RBC pellets. Total RNA isolation and cDNA synthesis was performed as illustrated previosuly^91^. Prior to microarray, the integrity of each RNA samples was checked on 1% agarose gel electrophoresis. cDNA samples derived from drug treated parasites were labeled with Cy5 and hybridized against the Cy3 labeled 3D7 reference pool. This common reference pool was obtained by assembling equal amounts of RNA from 6 time points corresponding to 8 hr interval stages throughout the IDC of the 3D7 strain.

### Microarray hybridization and data analysis

Identical amount of Cy5 and Cy3 labeled samples were hybridized to microarrays platform representing the *P. falciparum* genome. Our *Plasmodium* DNA array consists of 15,818 oligonucleotides probes (which includes 5,402 50-mer intergenic probes and 10,416 70-mer open reading frame probes) representing 5,343 protein coding genes^45^. The microarrays were scanned using GenePix scanner 400b and GenePix Pro 6.0 program (Axon Instruments). The raw microarray data obtained was subjected to lowess normalization as described^55^ and generates log transformed ratios of Cy5 intensity to Cy3 intensity. Hierarchical clustering was performed on these log-transformed ratios using Gene Cluster and results were visualized using Java Treeview.

### Chromatin immunoprecipitation

Chromatin immunoprecipitation was executed as explained previously^91^. Summarily, after 6 hr of MMS (0.05%) treatment at 3D7 trophozoite stage, parasites were harvested with 0.1% saponin. Isolated parasites were crosslinked with formaldehyde, lysed in 1% SDS buffer and homogenized using 200 strokes of dounce homogenizer. The derived nuclei pellet were sonicated with 8 pulses of 25% amplitude (10 sec pulse on, 50 sec off) in order to obtain DNA fragments in the range of 200–1000 bp. Sheared DNA was subjected to chromatin immunoprecipitation as described by Millipore ChIP Assay Kit (*#*17–295). Both immunoprecipitated DNA and input DNA (sonicated genomic DNA) from MMS treated and untreated samples was exponentially amplified using random primers 5′ GTTTCCCAGTCAGGAT-CNNNNNNNNN 3′ and 5′ GTTTCCCAGTCACGATC 3′ as described by^91^. For microarray, input was labeled with Cy 3 whereas immunoprecipitated DNA was labeled with Cy5. Input was made by mixing equal amount from MMS treated and untreated samples.

### Immunoblot analysis

Identical amount of total protein lysates obtained from drug treated samples and control/DMSO treated samples were separated by 15% SDS-PAGE. Following separation, proteins were transferred onto nitrocellulose membrane. Immunoblot analyses were carried out using primary antibodies probed against the core histone modifications obtained from Millipore, Upstate and horseradish peroxidase conjugated secondary antibody was acquired from GE Healthcare. Additionally, polyclonal PfHDAC1anti-serum, histone 4 (Millipore) and actin (Millipore) were used as loading controls.

### Quantitative real time PCR (RT-PCR)

Validations of microarray results for chosen genes were done by quantitative real time PCR (Roche) according to manufacturer instructions. Every cDNA sample was amplified in triplicates and to check specific amplification, no-template control was also included. Further, only those samples were included in data analysis, which shows single peak in melting curve. Relative fold change in RNA expression was computed by 2^−∆∆Ct^ method^92^. The reference control gene used was PF3D7_1218600 (arginyl-tRNA synthetase).

### High-throughput genome sequencing by Illumina MiSeq method

Genomic DNA was extracted from each of 3 parasites strains (Dd2, W2 and D6) and two microgram of genomic DNA was sequenced using whole-genome sequencing Illumina MiSeq method^64^. The sequenced data was aligned to the reference 3D7 *Plasmodium* genome. High quality SNP’s identified in the sequences using Genome Analysis Toolkit software (GATK) with stringent cut offs (minimum base quality score ≥ 30) as described by Mok *et al*.^93^. Using this cut-off, we identified 6,550, 7,231 and 7,253 non-synonymous SNP within coding regions in the Dd2, W2 and D6 strains, respectively.

## Additional Information

**Accession codes**: We have submitted the microarray data to NCBI GEO, (Accession number GSE72580).

**How to cite this article**: Gupta, D. K. *et al*. DNA damage regulation and its role in drug-related phenotypes in the malaria parasites. *Sci. Rep.* **6**, 23603; doi: 10.1038/srep23603 (2016).

## Supplementary Material

Supplementary Information

## Figures and Tables

**Table 1:**
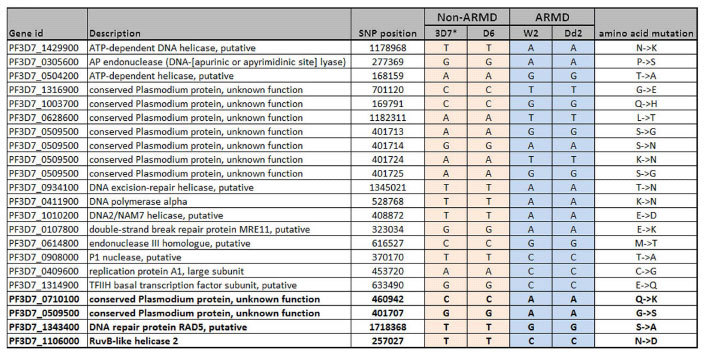
List of SNP located in DNA repair genes of ARMD parasites (Dd2 and W2).

These are 18 DNA repair genes which have non-synonymous SNP within coding region of ARMD parasites. Only one repair gene (PF3D7_0509500) have 4 SNP’s whereas remaining 17 genes have only one SNP per gene. SNPs located in four genes previously identified to be associated with artemisinin resistance in South East Asia are highlighted in red.*3D7 is reference genome, Rows highlighted with red are those ns-SNPs previously identified in artemisinin resistant samples.
