# Supplementary material for: DNA damage regulation and its role in drug-related phenotypes in the malaria parasites
Source: Sci Rep. 2016 Apr 1;6:23603. doi: 10.1038/srep23603 (PMC4817041; doi:10.1038/srep23603)
Supplement: Supplementary Information [file srep23603-s1.pdf]

DNA damage regulation and its role in drug-related phenotypes in the malaria parasites.

Devendra Kumar Gupta[1], Alok Tanala Patra[1], Zhu Lei[1], Archna Patkar Gupta[1] and Zbynek Bozdech[1,*]

[1] School of Biological Sciences, Nanyang Technological University, 639798, Singapore

* To whom correspondence should be addressed. Tel: (65) 63162925; Fax (65) 67913856;
Email:zbozdech@e.ntu.edu.sg

Supplementary figure 1

Pathways up-regulated by MMS (P value <0.05)

- Nucleotide excision repair
- Genes coding for enzymes involved in DNA replication
- Double strand break repair and homologous recombination
- Genes involved in excision-repair
- Base excision repair of AP sites
- Protein biosynthesis
- Initiation of translation
- Genes coding for components involved in ribosome assembly
- Nuclear genes with mitochondrial signal sequences
- Nuclear genes with apicoplast signal sequences
- Chaperone-assisted protein folding
- S-Glutathionylated proteins
- Glycolysis
- Pentose Phosphate Cycle
- Total palmitome of Plasmodium falciparum

DNA repair pathways (35 genes)

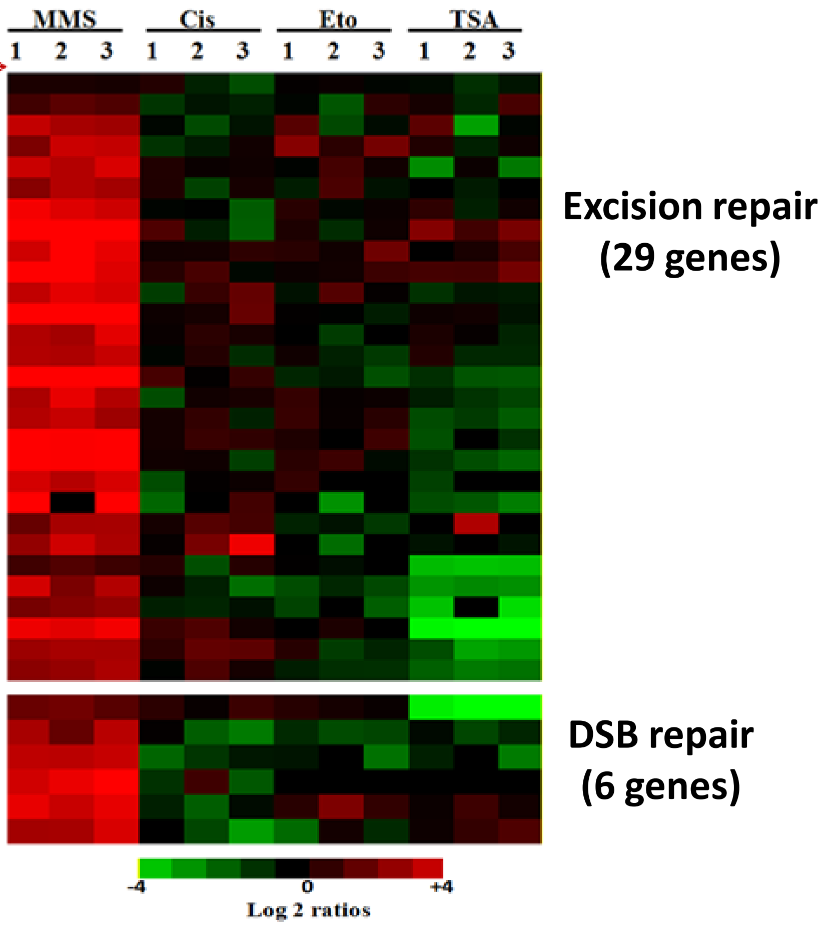

Supplementary figure 2

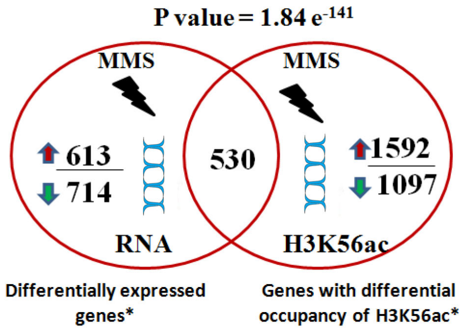

For H3K56ac, 189 genes (out of 530 overlapping) shows positive correlation between MMS induced RNA expression and H3K56ac

Enriched functional groups (p value < 0.05)

S-Glutathionylated proteins
Nuclear genes with mitochondrial signal sequences
Modification of proteins by adenylylation and ADP-ribosylation
Maturation and export of 60S and 40S ribosomal subunits
Genes coding for components involved in ribosome assembly
CoA biosynthesis
Chaperone-assisted protein folding
Asparagine and Aspartate metabolism

Supplementary figure 3

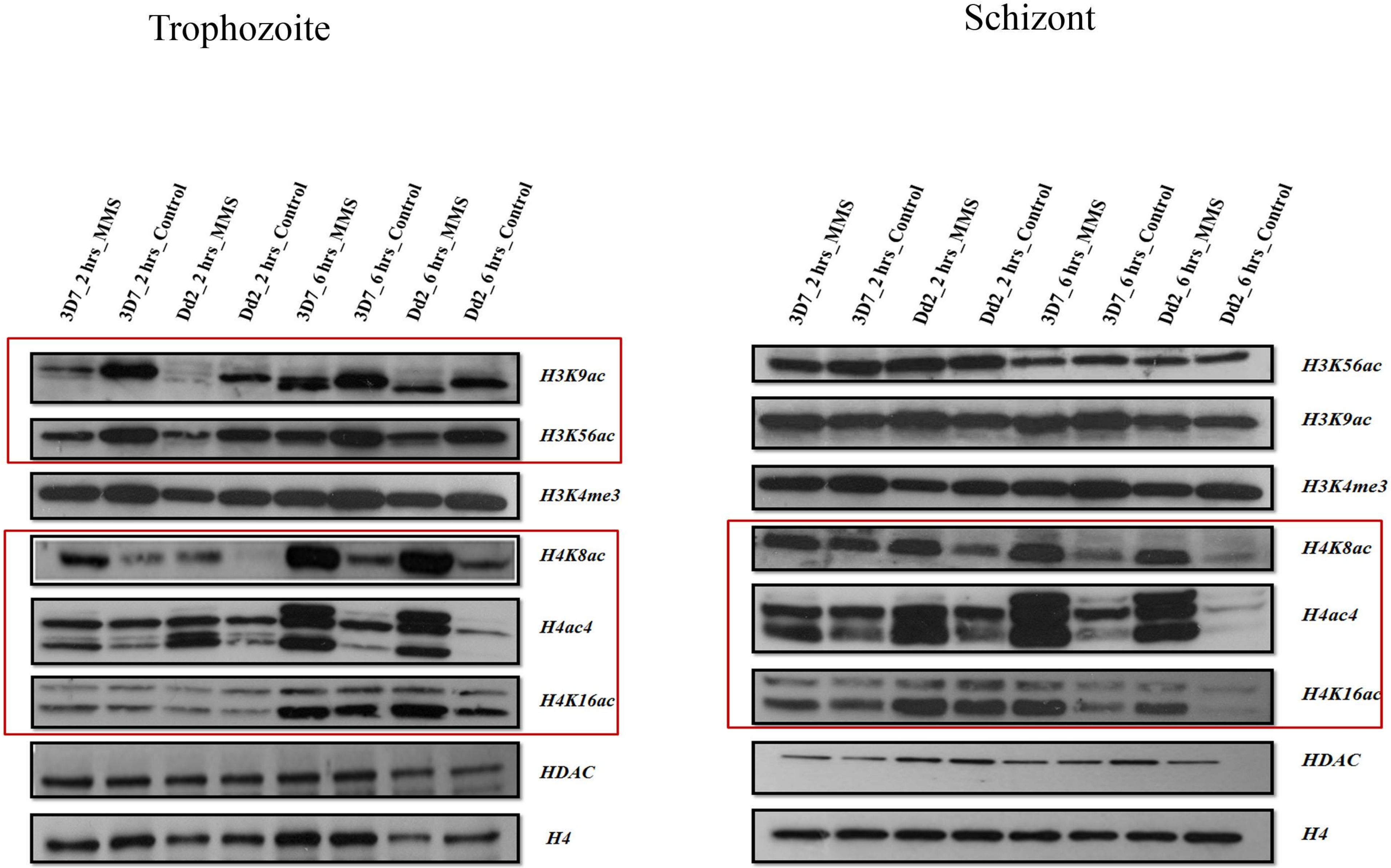

Table 1 SNP's identified in Dd2, W2 and D6 clones. Table shows the total no of single nucleotide polymorphism detected within Intron and exon (synonymous and non-synonymous SNP's) among D6, Dd2 and W2. The reference genome is 3D7. (*ns-SNP is non synonym single nucleotide polymorphism, **s-SNP is synonym single nucleotide polymorphism)

	Dd2	W2	D6
Intron-SNP	1523	1628	1497
Exon-s-SNP	2900	2812	2973
Exon-ns-SNP	**6550 (2099 genes)**	**6725 (2088 genes)**	**6278 (1942 genes)**
Total Intra-SNP	10973	11165	10748

Reference genome : 3D7 Map score > 30

Table 2 **SNP's identified in DNA repair genes among Dd2, W2 and D6 clones.** These are 57 DNA repair genes which have non-synonymous SNP within coding region. The reference genome is 3D7.

Gene ID	Description	Dd2	W2	D6
PF3D7_0726300	DNA mismatch repair protein PMS1, putative	18	13	4
PF3D7_1244200	transcription factor Tfb2, putative	11	11	10
PF3D7_1303800	conserved Plasmodium protein, unknown function	7	6	2
PF3D7_1368800	DNA repair endonuclease, putative	7	6	8
PF3D7_1368800	DNA repair endonuclease, putative	6	6	8
PF3D7_1037000	DNA polymerase zeta catalytic subunit, putative	5	5	5
PF3D7_0509500	conserved Plasmodium protein, unknown function	5	5	0
PF3D7_0514100	ATP-dependent DNA helicase UvrD	5	5	5
PF3D7_0310500	DEAD box helicase, putative	4	4	3
PF3D7_0504200	ATP-dependent helicase, putative	4	4	0
PF3D7_0605800	DNA repair protein RAD50, putative	4	2	12
PF3D7_0820000	Snf2-related CBP activator, putative	3	3	2
PF3D7_1455300	conserved Plasmodium protein, unknown function	3	3	1
PF3D7_0910500	DNA repair protein REV1, putative	3	3	2
PF3D7_1316900	conserved Plasmodium protein, unknown function	2	2	0
PF3D7_0710400	nucleotide excision repair protein, putative	2	2	2
PF3D7_1363500	DNase I-like protein, putative	2	2	1
PF3D7_1449700	exosome complex exonuclease RRP6, putative	2	2	2
PF3D7_1471600	conserved Plasmodium protein, unknown function	2	2	3
PF3D7_0204800	3'-5' exonuclease, putative	2	2	1
PF3D7_0409600	replication protein A1, large subunit	2	2	0
PF3D7_0411900	DNA polymerase alpha	2	2	0
PF3D7_0513600	deoxyribodipyrimidine photo-lyase, putative	2	2	9
PF3D7_0630300	DNA polymerase epsilon, catalytic subunit a	2	2	3
PF3D7_0908000	P1 nuclease, putative	2	2	0
PF3D7_1250800	DNA repair protein rhp16, putative	2	2	3
PF3D7_1331100	DEAD box helicase, putative	1	1	2
PF3D7_1343400	DNA repair protein RAD5, putative	1	1	0
PF3D7_1304100	DNA ligase I	1	1	1
PF3D7_1362500	exonuclease, putative	1	1	3
PF3D7_1314900	TFIIH basal transcription factor subunit, putative	1	1	0
PF3D7_0710100	conserved Plasmodium protein, unknown function	1	1	0
PF3D7_0803400	DNA repair protein RAD54, putative	1	1	1
PF3D7_1003700	conserved Plasmodium protein, unknown function	1	1	0
PF3D7_1010200	DNA2/NAM7 helicase, putative	1	1	0
PF3D7_1106000	RuvB-like helicase 2	1	1	0

PF3D7_1332100	conserved Plasmodium protein, unknown function	1	1	2
PF3D7_1337300	exoribonuclease, putative	1	1	1
PF3D7_1411400	plastid replication-repair enzyme	1	1	2
PF3D7_1429900	ATP-dependent DNA helicase, putative	1	1	0
PF3D7_1430600	exodeoxyribonuclease III, putative	1	0	0
PF3D7_1432600	conserved Plasmodium protein, unknown function	1	1	1
PF3D7_1449700	exosome complex exonuclease RRP6, putative	1	2	2
PF3D7_0107800	double-strand break repair protein MRE11, putative	1	1	0
PF3D7_0111300	replication factor c protein, putative	1	1	1
PF3D7_0206000	DNA repair endonuclease, putative	1	1	1
PF3D7_0216000	DEAD/DEAH box helicase, putative	1	1	2
PF3D7_0305600	AP endonuclease , putative	1	1	0
PF3D7_0416400	histone acetyltransferase, putative	1	1	3
PF3D7_0505500	DNA mismatch repair protein MSH6, putative	1	1	2
PF3D7_0604600	DNA helicase, putative	1	1	1
PF3D7_0614800	endonuclease III homologue, putative	1	1	0
PF3D7_0628600	conserved Plasmodium protein, unknown function	1	1	0
PF3D7_0917100	N-glycosylase/DNA lyase, putative	1	1	3
PF3D7_0934100	DNA excision-repair helicase, putative	1	1	0
PF3D7_1215700	conserved Plasmodium protein, unknown function	1	1	1
PF3D7_1235900	pre-mRNA-splicing factor SYF1, putative	1	1	5

Table 3 Comparison of DNA damage sensors among mammals, yeast and *P falciparum*.

DNA excision repair

Double strand break repair

Direct repair

Repair Mechanisms	Mammals	Yeast	*Plasmodium falciparum*	No of homologues in *P.falciparum*	MMS induced upregulation in *P.falciparum**
Base Excision (BER)	DNA glycosylase	DNA glycosylase	PF3D7_1129500, PF3D7_1415000, PF3D7_1467100, PF3D7_0917100	4	PF3D7_1129500, PF3D7_0917100
Nucleotide Excision (NER)	XPC-hHR23B complex	Rad4-Rad-23 complex	PF3D7_1432600 (XPC)<br>PF3D7_1011700 (Rad23)	XPC-1<br>Rad23-1	PF3D7_1432600 (XPC)<br>PF3D7_1011700 (Rad23)
Mismatch repair (MMR)	hMutSα (MSH2-MSH6)<br>hMutSβ (MSH2-MSH3)	MSH1-MSH3, MSH6	PfMSH2-1(PF3D7_1427500), PfMSH2-2 (PF3D7_0706700). PfMSH6 (PF3D7_0505500)	MSH2-2<br>MSH6-1<br>MSH3-Absent	PfMSH2-1(PF3D7_1427500)
Homologous Recombination (HR)	MRE11-RAD50-NBS1 (MRN complex)	Mre11-Rad50-Xrs2 (MRX complex)	PF3D7_0107800(MRE11)-PF3D7_0605800 (Rad50)	MRE11-1<br>PfRAD50-1<br>NBS1-Absent	PF3D7_0107800(MRE11)-PF3D7_0605800 (Rad50)
Non homologous recombination end joining (NHEJ)	Ku 70/80 complex	Ku 70/80 complex	Alternative NHEJ	Absent	
Single strand break (SSBR)	Replication binding protein -1(RPA-1)	Rfa1	PF3D7_0409600, PF3D7_0904800	2	PF3D7_0409600

AlkBH	AlkB homologue family *	AlkB homologue family *	ND	ND	
DNA photolyase (UV induced photo reactivation repair)	Absent in placental mammals	DNA photolyases (Phr1)	PF3D7_0513600 (DNA photolyase)	1	PF3D7_0513600
O 6Methylguanine-DNA methyltransferase (MGMT)	MGMT	MGMT	ND	ND	

ND - Not defined, * genes significantly up-regulated (p value < 0.05)

Table 4 Mapping of parasite age, hpi (hours post invasion) compared to *in vitro* IDC transcriptome of Dd2.

Samples	Maximum Correlation	Time Point
Cisplatin_replicate 1_post 6 hours	0.64	38 hpi
Cisplatin_replicate 2_post 6 hours	0.73	38 hpi
DMF_replicate 1_post 6 hours	0.75	38 hpi
DMF_replicate 2_post 6 hours	0.75	38 hpi
Etoposide_replicate 1_post 6 hours	0.73	40 hpi
Etoposide_replicate 2_post 6 hours	0.72	38 hpi
DMSO_replicate 1_post 6 hours	0.76	38 hpi
DMSO_replicate 2_post 6 hours	0.75	38 hpi
TSA_replicate 1_post 6 hours	0.59	38 hpi
TSA_replicate 2_post 6 hours	0.58	38 hpi
Control for MMS_replicate 1_post 6 hours	0.83	38 hpi
Control for MMS_replicate 2_post 6 hours	0.84	38 hpi
MMS_replicate 1_post 6 hours	0.55	30 hpi
MMS_replicate 2_post 6 hours	0.56	30 hpi

DMF is vector control for cisplatin, DMSO is vector control for etoposide and TSA. MMS is dissolved in RPMI.

Supplementary figure 1. **Upregulation of DNA damage repair genes in MMS treated *P. falciparum.*** Functional enrichment analysis of genes upregulated by 6 hr treatment with 0.05% MMS. The Gene Set Enrichment Analysis (GSEA) was used to identify the functional pathways enriched amongst the sets of upregulated genes with statistical significance P value <0.05.The heat map represents relative mRNA abundance values for 29 and 6 genes involved in excision and DSBR in the *P. falciparum* cells treated with the four DNA damaging agents. (For details, see materials and methods section.)

Supplementary figure 2. **Correlation analysis between MMS induced gene expression and H3K56ac.** The Venn diagram depicts the overlap between the 1327 genes were differentially expressed (714 down and 613 up-regulated) and 2689 genetic loci (corr. microarray probes) with differential occupancy of H3K56ac as a result of MMS (P value <0.05). The 530 genes showed differential expression and H3K56ac occupancy which accounts for a statistically overlap; P value 1.84e-141. From these, 189 genes showed increased expression and increased H3K56ac occupancy. The list indicates functional pathways enriched in the set of 189 genes (P value <0.05).

Supplementary figure 3.**Western blot analysis of histone modifications abundance in ARMD and non-ARMD *P. falciparum* strains.** The ARMD (Dd2) and non-ARMD (3D7) strains of *P. falciparum* were treated with MMS (0.05%) for 2hr and 6hr at two developmental stages (trophozoite and schizont). The changes in the overall abundance of histone marks were monitored in the total cell lysates using specific antibody in western blot analysis. Specific antibody against HDAC and H4 were used as loading controls.
